# How we assess the perioperative anxiety of surgical patients with pulmonary nodules: the revision of state-trait anxiety inventory

**DOI:** 10.1186/s13019-020-01338-1

**Published:** 2020-10-28

**Authors:** Zhenyu Zhou, Ying Wang, Yuequn Niu, Zhehao He, Manli Huang, Yuqiong Zhou, Wang Lv, Jian Hu

**Affiliations:** 1 Department of Thoracic Surgery, the First Affiliated Hospital, School of Medicine, Zhejiang University, Hangzhou, 310003 China; 2 Operation Room, the First Affiliated Hospital, School of Medicine, Zhejiang University, Hangzhou, 310003 China; 3 Department of Psychiatry, the First Affiliated Hospital, School of Medicine, Zhejiang University, Hangzhou, 310003 China

**Keywords:** State-trait anxiety inventory, Surgical patients, Perioperative anxiety, Structural equation model

## Abstract

**Purpose:**

The aim of the study was to develop a short form of State-Trait Anxiety Inventory (STAI) and calculate the norms for the assessment of anxiety in surgical patients in mainland China.

**Methods:**

Patients who were scheduled to carry out pulmonary surgery in our department were included. The sinicized 40-item STAI Form-Y was used to assess the anxiety on the surgery eve. Then the coefficient of variation, coefficient of correlation, stepwise regression analysis, principal component analysis, and structural equation model were successively to filter the items. The reliability and validity of the revised STAI was estimated and the norms were computed.

**Results:**

445 intact replies were collected. A 13-item STAI with 6 items in state subscale and 7 items in trait subscale produced similar scores with the full version of STAI. The Cronbach alpha coefficients for the state and trait subscales were 0.924 and 0.936, respectively. The determinant coefficients were 0.781 and 0.822, respectively. Moreover, the norms of both state subscale and trait subscale are provided according to the age and gender.

**Conclusions:**

The revised short form of STAI has good reliability and validity. It is likely to be more acceptable by reducing the fatigue effects, and is suitable for follow-up study on the assessment and intervention of perioperative anxiety of surgical patients with pulmonary nodules.

## Introduction

Lung cancer is still the most widespread and important malignant tumor at present, since it attributes over one eighth to the morbidity and nearly one quarter to the mortality in all malignant tumors [1]. Surgery is still the most effective treatment for patients with early-stage lung cancer [2–6]. At the beginning of twenty-first century, the concept of enhanced recovery after surgery (ERAS) emphasizes the integrated application of various methods to enhance perioperative management and promote postoperative recovery of patients as well, including pain relief, minimally invasive operation, and so on [7–11]. The assessment and intervention of perioperative anxiety should also be a part, and it is getting more and more attention [12–16].

Our department intends to conduct a registry study to assess and intervene perioperative anxiety in patients undergoing pulmonary surgery. To this end, we asked the mental health specialists to help us develop a comprehensive preoperative relaxation training process including progressive muscle relaxation training and breathing relaxation training [2, 14, 17–19]. At the same time, by consulting the literature, we selected the commonly used State-Trait Anxiety Inventory (STAI) as an indicator to assess perioperative anxiety.

Form Y, the most popular version of STAI, is a self-report, 40-item psychological test for adults, and it is divided into a State Anxiety Inventory (From Q01 to Q20) and a Trait Anxiety Inventory (From Q21 to Q40). Each item is rated on a 4-point Likert scale [20]. The state anxiety, which is an immediate and unstable emotional state, is assessed by the rating of his or her temporary feelings at the particular moment. And the trait anxiety, which is a relatively stable and enduring personality characteristic, is assessed by the rating of his or her general feelings across time [21]. The inventory has been reported to be suitable for use in clinical settings to assess the anxiety levels of unwell and healthy participants [14, 22–24]. And it has also been translated to various languages including Chinese.

However, in the earlier small sample survey, we found that patients of different ages and different genders showed grate difference in preoperative anxiety. And STAI, as a 40-item inventory, was reported to be too complicated by several participants, similar as previous studies [23]. Thus, we conducted this pilot study to identify the population to participate the following study and to develop a revised version of STAI for use in the following study [25]. There are two main objectives in this study:

(i) to illustrate the gender and age group who have higher level of preoperative anxiety;
(ii) to revise the STAI Form-Y with less items to assess the preoperative anxiety of surgical patients with pulmonary nodules equally effectively.

## Materials and methods

### Patients selection

From Aug 1st, 2018 to Jul. 31st, 2019, we filter the patients who were scheduled to carry out pulmonary surgery due to the suspected or confirmed lung cancer in our department.

The inclusion criteria were: No less than 18 years old (the standard for adults in mainland China); the patient’s ability to directly communicate with medical staffs; the patient’s informed consent (with no objection of family members); no history of pulmonary surgery.

The exclusion criteria were: Less than 18 years old (minors); planning to carry out other operations besides pulmonary surgery; no direct or smooth communication between the patient and medical staffs; refused by the patient or family members; involved in previous related researches; involved in other studies during this hospital stay; withdrawn at any time; other situations in which the patient is considered unable to complete the study.

### Materials

We selected the State-Trait Anxiety Inventory (STAI) Form-Y to measure the anxiety of the patients. The inventory has respective norms for state anxiety and trait anxiety in different gender and age groups. A score higher than the norm is considered to be positive. We have published the online version of the inventory according to the Chinese version, which can be easily assessed by scanning the QR code or clicking the link using a smartphone Appendix 1.

### Procedure

The research team consists of thoracic surgeons, mental health specialists, and operation room nurses. All members of the team are not the medical staffs directly responsible for the patients during the treatment. Firstly, surgeons collected information about the patients to be admitted for pulmonary surgery. Secondly, nurses contacted the patients in advance and sought their informed consent. On the surgery eve, when preoperative conversation and preparation were completed, our nurses would present the QR code or link to the patients and ask for their own answers. If the patient has reading or literacy difficulties, the staff would read out the contents of the inventory without inclination, and then the patient could answer by himself.

### Statistical analysis

The significance level of all statistical analysis was set at *p* < 0.05. Firstly, identify the excluded and reserved items. In IBM SPSS Statistics 25.0 (IBM, Armonk, New York, USA), the coefficient of variation (CV) was used to exclude the items whose CV was less than 0.250. The correlation coefficient (CC) method was used to exclude the items whose Spearman’s rank correlation coefficient (R) with the subscale was less than 0.60. The stepwise regression analysis was used to exclude the items which were not included in the regression analysis model. The principal component analysis (PCA) was used to excluded the items whose factor loadings were less than 0.500 on each factor or were close on two or more factors in the largest variance rotation model. In IBM SPSS Amos 26.0 (IBM, Armonk, New York, USA), the confirmatory factor analysis was carried out to establish a structural equation model and then modify the model in order to get the reserved items of the revised STAI.

Structural equation model (SEM) is a confirmatory factor analysis, which is used to explore the correlation between independent variable (item score) and dependent variable (subscale score). Chi square (χ^2^), Goodness-of-Fit Index (GFI) and Root Mean Square Error of Approximation (RMSEA) are important fitting indexes of SEM. The larger the GFI is, and the smaller the χ^2^ and RMSEA are, the better the fitting degree of SEM is. Item filtering started from the item whose regression weight with the subscale score was the smallest. If the SEM fitting is better after the deleting of an item, the item was confirmed to be deleted. While if the SEM fitted worse after the deleting, the item would be retained. It would be repeated until the best SEM fitting was achieved. At this point, the items which were still kept in the SEM were the ones we would keep for the revised STAI.

Secondly, estimate the reliability and validity of the revised STAI. Cronbach α was used to evaluate the internal consistency. The decision coefficient, *r*^2^, of the scores of revised STAI and the original STAI Form-Y was calculated using correlation analysis. And the Youden index of revised STAI, which is 1.000 less than the sum of sensitivity and specificity, was calculated. A Youden index close to 0 indicates that the accuracy of the inventory is poor, while a Youden index close to 1 indicates that the accuracy is perfect.

Thirdly, compute the norm of the revised STAI through the linear regression analysis with the original norm.

At last, determine the appropriate patient groups for the following study according to the positive rate of perioperative anxiety in different age and gender groups.

## Results

### Demography

**Fig. 1:**
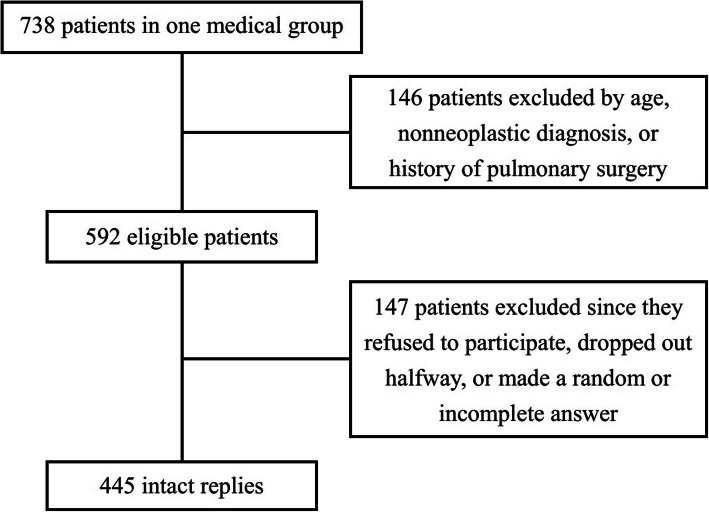
The Flow Chart of Patients Selection

We distributed the inventory to 592 eligible patients, and then collected 445 intact replies. The response rate was 75.2% (Fig. 1). The demographic characteristics of the participants are shown in Table 1. All the patients would undergo a radical resection of the suspected or confirmed lung cancer.

**Table 1:** The Demographic Characteristics of Participants

	No.	%
Gender		
Male	226	50.8
Female	219	49.2
Age Group		
18 and below	0	–
19–39	41	9.2
40–49	76	17.1
50–60	148	33.3
61 and above	180	40.4

### The revised STAI

No items were excluded due to the CV, and no items were excluded by the regression analysis model. In the CC statistics, 4 items (Q01, Q07, Q12 and Q14) in State subscale (S-AI) and 10 items (Q24, Q25, Q28, Q29, Q31, Q32, Q35, Q37, Q38 and Q40) in Trait subscale (T-AI) were excluded due to the low value of R. In PCA statistics, the KMOs of S-AI and T-AI were 0.942 and 0.943. That means the set of data is available for PCA. Then no more items were excluded.

**Fig. 2:**
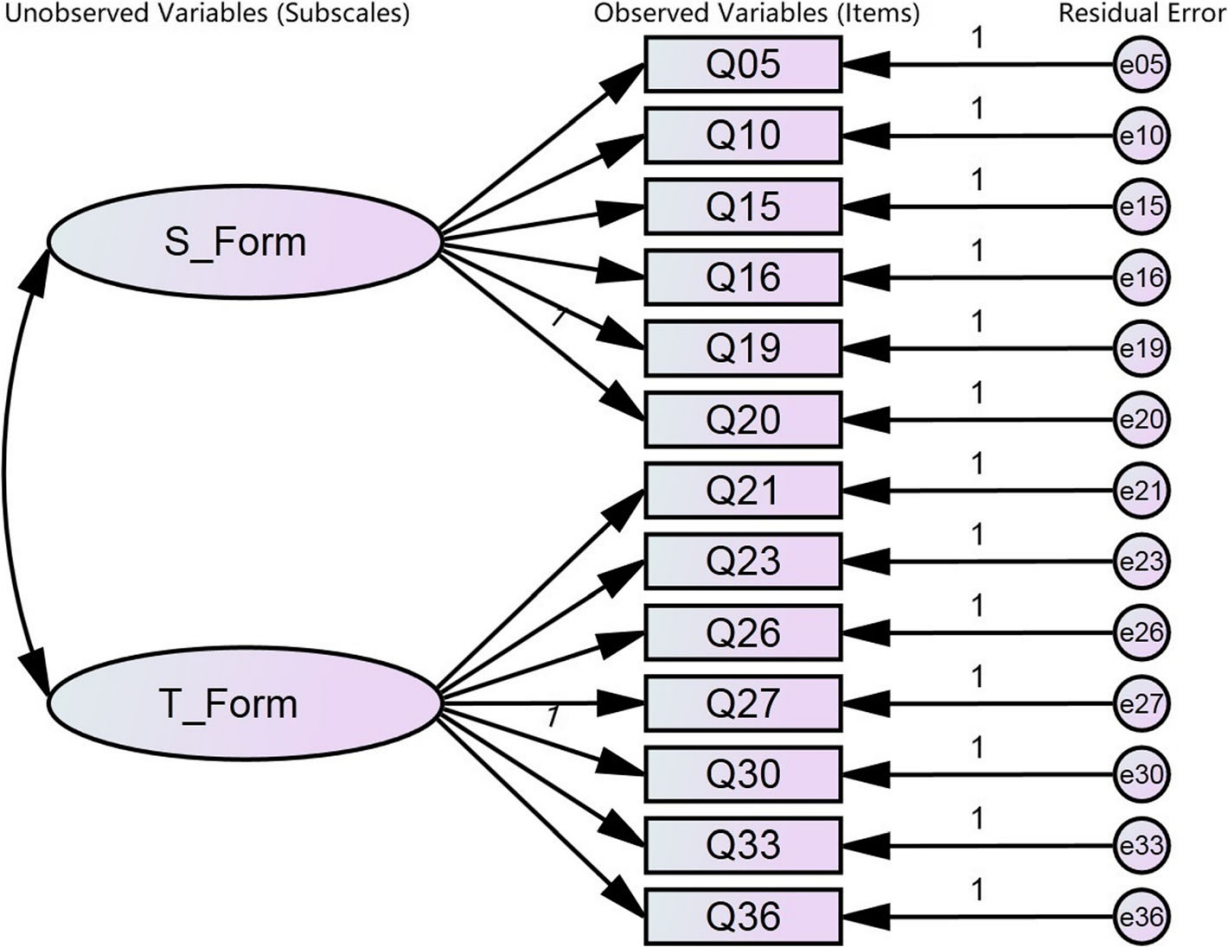
The Structural Equation Model

Input the left 16 items of S-AI and 10 items of T-AI into the structural equation model. After calculation, a revised 13-item STAI was obtained with a 6-item S-AI (Q05, Q10, Q15, Q16, Q19, Q20) and a 7-item T-AI (Q21, Q23, Q26, Q27, Q30, Q33, Q36). The structural equation model is shown in Fig. 2.

The Cronbach α of S-AI and T-AI are 0.924 and 0.936. The *r*^2^ of S-AI and T-AI are 0.781 and 0.822. The sensitivity, specificity and Youden index of S-AI are 0.867, 0.927 and 0.794, and those of T-AI are 0.813, 0.912 and 0.725.

The norm of the revised STAI is shown in Table 2. The positive rates of perioperative anxiety in different age and gender groups are shown in Tables 3 and 4.

**Table 2:** The Norm of Revised STAI

	19–39		40–49		50–60	
	Male	Female	Male	Female	Male	Female
S-AI	21	23	22	24	20	18
T-AI	23	25	24	26	23	20

*STAI* State-Trait Anxiety Inventory, *S-AI* State Anxiety Inventory, *T-AI* Trait Anxiety Inventory

**Table 3:** The Positive Rates (%) of Perioperative Anxiety

	19–39		40–49		50–60	
	Male	Female	Male	Female	Male	Female
S-AI	9.1	20.3	10.3	6.4	11.7	45.1
T-AI	27.3	13.3	17.2	6.4	10.4	49.3
STAI	27.3	33.3	17.2	10.6	15.6	57.7

*STAI* State-Trait Anxiety Inventory, *S-AI* State Anxiety Inventory, *T-AI* Trait Anxiety Inventory

**Table 4:** The items retained in 13-item version of STAI

Below are some of the statements that people often use to describe themselves. Read each statement and tick the appropriate circle on the right to indicate your most appropriate feeling at the moment. There is no right or wrong answer. Don’t spend too much time thinking about any statement, but the answer should be your most appropriate feeling now.					
	Here’s how you feel at the moment.	Not at all	Somewhat	Moderately so	Very much so
05	I feel at ease*	①	②	③	④
10	I feel comfortable*	①	②	③	④
15	I am relaxed*	①	②	③	④
16	I feel content*	①	②	③	④
19	I feel steady*	①	②	③	④
20	I feel pleasant*	①	②	③	④
	Here’s how you feel often.	Not at all	Somewhat	Moderately so	Very much so
21	I feel pleasant*	①	②	③	④
23	I feel satisfied with myself*	①	②	③	④
26	I feel rested*	①	②	③	④
27	I am ‘calm, cool and collected’*	①	②	③	④
30	I am happy*	①	②	③	④
33	I feel secure*	①	②	③	④
36	I am content*	①	②	③	④

Reverse counting items are shown with *

## Discussion

The primary objective of the study was to develop a concise version of STAI which would be suitable for the assessment of perioperative anxiety in pulmonary surgery patients. Overall, the revised 13-item STAI has a good internal consistency and its structure is substantially consistent with the original STAI Form-Y. In addition, the application of the revised STAI will have some advantages over the original version.

Firstly, the original 40-item STAI was reported as being too long by some of the participants. And there were also some participants making answers indiscriminately, which made his reply invalid. We found that the time taken for a reply was extremely different, ranging from 1 min 40 s to 58 min 18 s, with a median of 6 min 40 s, and quartiles of 4 min 54 s and 9 min 41 s. One possible reason is that an inventory with too many items will consume too much time and effort of the participant. It would affect the compliance of some of the participants, especially those not very concerned about the study. The response rate would also be affected by the number of items.

Secondly, the response requires a period of time. While the patients need much time for preoperative accessory examination, preoperative communication and signature, preoperative preparation, and so on. A concise inventory would make it easier for the participants schedule the time without interfering with other arrangements.

Finally, the revised STAI still contains the two subscales of S-AI and T-AI, with good reliability and validity. It will not affect the follow-up study to distinguish the clinical characteristics of patients with different levels and types of anxiety.

The study also has some limitations that should be acknowledged. Firstly, this study is a single-center study. While, our department ranked 1st in Zhejiang Province and 10th in mainland China in 2017, and our hospital ranked 1st in Zhejiang Province and 10th in mainland China in 2018. It should be said that the participants in this study are well-represented in East China. However, it cannot be ruled out that the participants in this study may have different clinical and psychological characteristics from the ones in other regions of the country, due to the regional, economic and other reasons. But as a pilot study, the results are still sufficient for the follow-up study.

In addition, the clinical and social characteristics of the participants, including the lesion size, the resection range, preoperative diagnosis, patients’ awareness of the condition, history of surgery with general anesthesia, education level, family structure, family income, etc., were not distinguished in this study. These contents need further research.

Patients in different age and gender groups have different positive rates of perioperative anxiety. According to the result, the follow-up study will be conducted on female patients between 50 and 60 years old. And we will try to draw a norm of perioperative anxiety for female patients over 61 years old.

## Conclusions

In this study, a revised STAI was developed to assess perioperative anxiety in pulmonary surgery patients. The inventory has good reliability and validity, and is suitable for follow-up study. Lung cancer has a high incidence and mortality. It is a very meaningful work to carry out a study on the assessment and intervention of perioperative anxiety of pulmonary surgery patients.

## Data Availability

The datasets used during the current study are available from the corresponding author on reasonable request.

## Appendix

**Table 5:** State-Trait Anxiety Inventory

Below are some of the statements that people often use to describe themselves. Read each statement and tick the appropriate circle on the right to indicate your most appropriate feeling at the moment. There is no right or wrong answer. Don’t spend too much time thinking about any statement, but the answer should be your most appropriate feeling now.					
	Here’s how you feel at the moment.	Not at all	Somewhat	Moderately so	Very much so
01	I feel calm*	①	②	③	④
02	I feel secure*	①	②	③	④
03	I am tense	①	②	③	④
04	I feel strained	①	②	③	④
05	I feel at ease*	①	②	③	④
06	I feel upset	①	②	③	④
07	I am presently worrying over possible misfortunes	①	②	③	④
08	I feel satisfied*	①	②	③	④
09	I feel frightened	①	②	③	④
10	I feel comfortable*	①	②	③	④
11	I feel self-confident*	①	②	③	④
12	I feel nervous	①	②	③	④
13	I am jittery	①	②	③	④
14	I feel indecisive	①	②	③	④
15	I am relaxed*	①	②	③	④
16	I feel content*	①	②	③	④
17	I am worried	①	②	③	④
18	I feel confused	①	②	③	④
19	I feel steady*	①	②	③	④
20	I feel pleasant*	①	②	③	④
	Here’s how you feel often.	Not at all	Somewhat	Moderately so	Very much so
21	I feel pleasant*	①	②	③	④
22	I feel nervous and restless	①	②	③	④
23	I feel satisfied with myself*	①	②	③	④
24	I wish I could be as happy as other seem to be*	①	②	③	④
25	I feel like a failure	①	②	③	④
26	I feel rested*	①	②	③	④
27	I am ‘calm, cool and collected’*	①	②	③	④
28	I feel that difficulties are piling up so that I cannot overcome them	①	②	③	④
29	I worry too much over something that really doesn’t matter	①	②	③	④
30	I am happy*	①	②	③	④
31	I have disturbing thoughts	①	②	③	④
32	I lack self-confidence	①	②	③	④
33	I feel secure*	①	②	③	④
34	I make decision easily*	①	②	③	④
35	I feel inadequate	①	②	③	④
36	I am content*	①	②	③	④
37	Some unimportant thought runs through my mind and bothers me	①	②	③	④
38	I take disappointments so keenly that I can’t put them out of my mind	①	②	③	④
39	I am steady person*	①	②	③	④
40	I get in a state of tension or turmoil as I think over my recent concerns and interests	①	②	③	④

Reverse counting items are shown with *

## Abbreviations

STAI: State-Trait Anxiety Inventory
ERAS: Enhanced recovery after surgery
CV: Coefficient of variation
CC: Correlation coefficient
PCA: Principal component analysis
SEM: Structural equation model
GFI: Goodness-of-Fit Index
RMSEA: Root Mean Square Error of Approximation
S-AI: State subscale of STAI
T-AI: Trait subscale of STAI
